# Bio-Inspired Iron-Loaded Polydopamine Functionalized Montmorillonite as an Environmentally Friendly Flame Retardant for Epoxy Resin

**DOI:** 10.3390/molecules28145354

**Published:** 2023-07-12

**Authors:** Jiashui Lan, Dingsi Li, Wei Zhong, Wenhui Luo, Huagui Zhang, Mingfeng Chen

**Affiliations:** 1 Fujian Key Laboratory of Polymer Materials, College of Chemistry and Materials Science, Fujian Normal University, Fuzhou 350007, China; 2 Research and Development Department, Waexim (Xiamen) New Materials Co., Ltd., Xiamen 361023, China

**Keywords:** epoxy resin, iron-loaded polydopamine, montmorillonite, flame retardancy

## Abstract

As an important thermosetting material, flame-retardant epoxy resin has various applications in the aerospace, chemical, and electronics industry, and other fields. However, the flame retardancy of epoxy resins is often improved at the expense of mechanical performance. The contradiction between flame retardancy and mechanical properties seriously impedes the practical applications of epoxy resin (EP). Herein, iron-loaded polydopamine functionalized montmorillonite (D-Mt-Fe^3+^), which was prepared by dopamine, iron chloride and montmorillonite in an aqueous solution, was introduced to prepare iron-loaded polydopamine functionalized montmorillonite/epoxy resin composites (D-Mt-Fe^3+^/EP). As expected, D-Mt-Fe^3+^/EP-10 with 10 phr of D-Mt-Fe^3+^ passed the UL-94 V-0 rating, achieved a limiting oxygen index (LOI) value of 31.0% and reduced the smoke production rate (SPR) and total smoke production (TSP), indicating that the introduction of D-Mt-Fe^3+^ could endow EP with satisfactory flame retardancy through the radical scavenging function of dopamine in the gas phase and the catalytic charring effect of iron ions, respectively. Encouragingly, the mechanical property was also enhanced with the flexural strength increased by 25.5%. This work provided an attractive strategy for improving both the mechanical properties and fire resistance of EP, which greatly broadened their applications in the chemical industry and electronics field, etc.

## 1. Introduction

With the rapid growth of polymer materials, the flame-retardant performance of these polymers (such as epoxy resin, unsaturated polyester, etc.) and their fiber-reinforced composite materials, which are commonly used in the chemical industry and electronics field, has gradually received attention because the flammability poses a great threat to the safety of people’s lives and property in the case of various fire accidents [1]. As a thermosetting material, epoxy resin (EP) is widely used in coatings, adhesives, sealants and lightweight structures in automobiles and ships because of its good dimensional stability, strong corrosion resistance and enhanced mechanical properties [2]. In addition, epoxy resin also occupies a pivotal position in fiber-reinforced composite materials. With the development of the automotive and electronics industry, epoxy resin is in huge demand [3,4]. However, compared to other commonly used polymers, epoxy resins have poor flame-retardant properties [5]. For example, the limiting oxygen index of epoxy resin is only about 19 [6], which means that it will continue to burn once ignited in air. EP has the disadvantage of being highly flammable, which limits its widespread applicability in various fields.

Up to now, many chemical compounds have been discovered to be used as flame retardants to inhibit the spread of flames. According to the chemical structure, flame retardants can be divided into organic and inorganic flame retardants. For organic flame retardants, some halogen-containing flame retardants exhibit excellent flame-retardant properties. However, these halogen-containing flame retardants would release toxic and harmful gases when they burned, which can cause damage to humans and the environment. With the emphasis on environmental protection, halogenated flame retardants have been greatly limited [7,8]. Some organic compounds containing phosphorus, silicon and nitrogen show good flame-retardant properties and have attracted much attention [9,10,11,12]. However, these organic compounds also have some drawbacks. For example, micro-molecule liquid organic flame retardants can easily penetrate out of the polymer matrix due to their weak interaction with the polymer matrix, resulting in poor flame retardancy [13]. In addition, the synthesis process of macro-molecule organic flame retardants is usually complex with low yields. Up to now, inorganic flame retardants are often used in flame retardant EP because of their smokeless, non-toxic and inexpensive characteristics [14,15,16]. As a kind of inorganic filler, montmorillonite (MMT) has the advantages of small size, high thermal stability and high intercalation capacity [17], which has received special attention in the field of flame retardants [18,19]. MMT can generate SiO_2_ and other substances to cover the surface layer during the combustion process, playing a role in protecting the underlying resin matrix from further damage [20,21]. Meanwhile, MMT can also help to reduce the release rate of smoke, heat, and toxic gases during the combustion process [22,23]. However, due to the agglomeration and hydrophilic properties of MMT, the interaction between EP and MMT is weak, which deteriorates the mechanical properties of the modified EP and has become a major limitation for further application.

Currently, surface treatment is the most commonly used method for enhancing the interaction between EP and MMT [24,25,26]. For MMT, a suitable coupling agent is usually used to reduce its surface polarity and thus improve the compatibility with epoxy resins. Kim et al. used 3-aminopropyltriethoxysilane (3-APTES) for the surface treatment of MMT to reinforced epoxy composites [27]. Batool et al. prepared epoxy multilayer composites using (3-aminopropyl) trimethoxysilane (APTMS)-treated MMT [28]. Hua et al. used (3-aminopropyl)-tri-ethoxy-silane (APTES) as a coupling agent to couple graphene oxide with nano-MMT. The polar effect of the APTES group helped to improve the dispersion of the nanomaterials in the epoxy-resin coating [29]. Nevertheless, with increasing concern for environmental protection, a great deal of research has turned to bio-based materials for the modification of MMT. Among them, polydopamine (PDA) has received widespread attention as a new and efficient biomass modifier [30,31]. The structure of a dopamine (DA) molecule is similar to the secreted mussel adhesion protein catechol, and its surface is rich in active amino groups and catechol. Under alkaline conditions, polydopamine membranes rich in active groups can be spread on the surfaces of various materials by oxidative self-polymerization in air [32,33]. In addition, the amino and catechol groups on the surface of dopamine can be used as reaction sites for metal ions. The addition of transition metal ions, such as Fe^3+^, Mn^3+^ and Ni^2+^, to the dopamine polymerization reaction can lead to the formation of transition metal-containing PDA materials [34,35,36]. Metal-containing dopamine materials have a stronger ability to scavenge radicals than pure PDA [37], especially iron-loaded polydopamine (Fe-PDA), which can act as catalysts for the charring of EP materials during combustion [38]. And the radical scavenging ability of the PDA can also improve the flame retardancy of EP [39,40].

**Figure 1:**
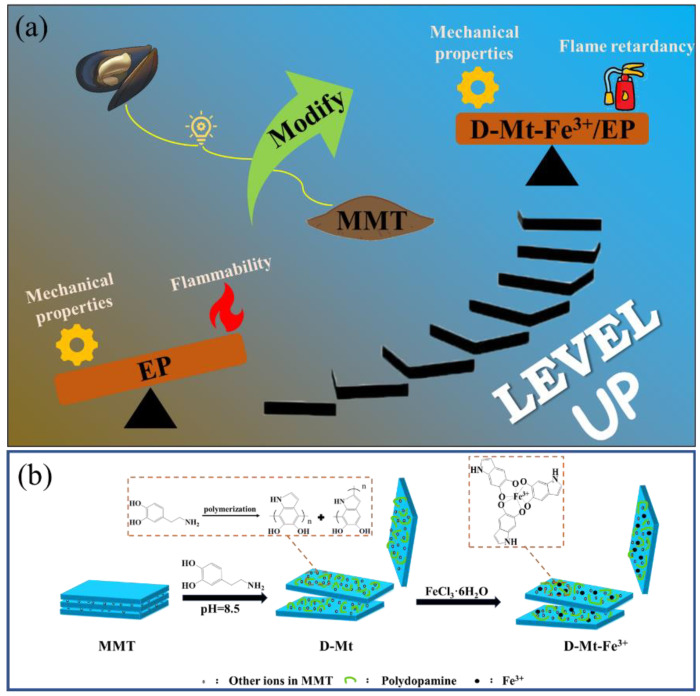
(**a**) Design of D-Mt-Fe^3+^/EP, and (**b**) preparation of D-Mt-Fe^3+^.

Herein, in order to realize a dual performance improvement simultaneously in epoxy resin, we propose a novel iron-loaded polydopamine functionalized montmorillonite (D-Mt-Fe^3+^), which can be obtained by the treatment of MMT with biomass dopamine complexed with iron ions. The obtained D-Mt-Fe^3+^ was confirmed by FTIR and XPS. After that, D-Mt-Fe^3+^ was added to epoxy resin to prepare iron-loaded polydopamine functionalized montmorillonite/epoxy resin composites (D-Mt-Fe^3+^/EP), as shown in Figure 1a. The adhesion and radical-scavenging abilities of PDA are expected to help improve the dispersion and flame-retardant efficiency of MMT. In addition, a detailed analysis of char residue after a cone calorimeter test is conducted to investigate the catalytic carbonization ability of D-Mt-Fe^3+^ in EP composites. The flame retardancy of D-Mt-Fe^3+^ in the gas phase and the condensed phase is also summarized. As expected, the resulting D-Mt-Fe^3+^/EP has improved mechanical properties while reducing the risk of fire.

## 2. Results and Discussion

### 2.1. Structural Analysis of D-Mt-Fe^3+^

**Figure 2:**
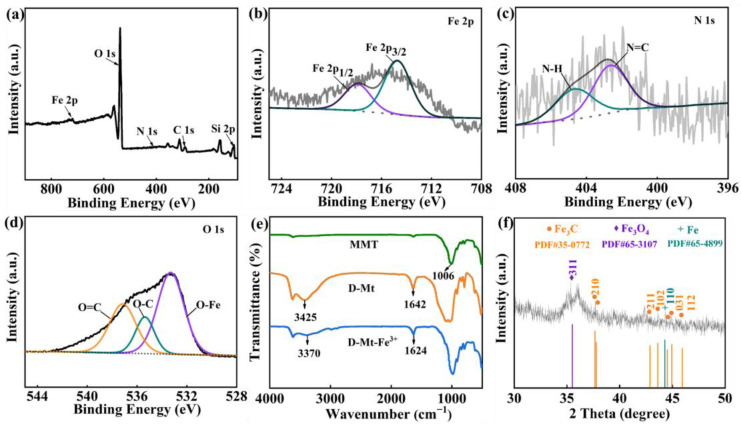
(**a**–**d**) XPS, (**e**) FTIR spectra of D-Mt-Fe^3+^ and (**f**) XRD spectra of residual carbon of D-Mt-Fe^3+^/EP.

The element components in D-Mt-Fe^3+^ were analyzed by XPS, and the presence of Fe, O, N, C and Si in D-Mt-Fe^3+^ can be observed in Figure 2a, indicating that the preparation of D-Mt-Fe^3+^ was successful. The elemental states in D-Mt-Fe^3+^ were further analyzed. The spectrum of Fe 2p (Figure 1b) was divided into two main peaks with binding energies at 717.9 eV and 714.7 eV, corresponding to Fe 2p_1/2_ and Fe 2p_3/2_, respectively, which indicate that the iron in D-Mt-Fe^3+^ is present mainly in the ferric state, not ferrous state. Two distinct peak intensities at 404.6 eV and 402.6 eV were mainly due to N–H and N=C, respectively, indicating D-Mt-Fe^3+^ carries a large number of amino groups, which is favorable for the interaction between the synthesized product and the EP substrate (Figure 2c). The deconvolution of the O 1s peak in Figure 2d showed O=C (537.2 eV), O–C (535.4 eV) and O–Fe (533.3 eV) signals. This shows that coordination bonds are formed between iron ions and catechol groups on the surface of D-Mt-Fe^3+^. Figure 2e shows the infrared spectra of MMT, D-Mt and D-Mt-Fe^3+^. The characteristic peak of MMT appeared at 1006 cm^−1^, which was attributed to the Si–O vibration. The IR spectrum of D-Mt reveals adsorption peaks at 1642 and 3425 cm^−1^, which can be attributed to the stretching vibration of aromatic ring and hydroxyl group, respectively. This indicates that the polydopamine was successfully bound to the MMT surface. In the IR spectrum of D-Mt-Fe^3+^, a phenolic peak at 3370 cm^−1^ appeared which was weaker than the peak at 3425 cm^−1^ of D-Mt, which is due to the complexation of the iron ion with the catechol on the polydopamine [41]. Furthermore, it can be seen from XRD spectra (Figure 2f) that various Fe-containing substances such as Fe_3_O_4_, Fe_3_C and Fe were formed after combustion. The Fe^3+^ in the D-Mt-Fe^3+^ can react with nearby hydrocarbons during combustion to form Fe_3_C. In the presence of oxygen and the pyrolytic gasification products of the epoxy resin, the Fe^3+^ can be oxidized to form Fe_3_O_4_ with a partial reduction to Fe [42].

### 2.2. Thermal Stability

**Figure 3:**
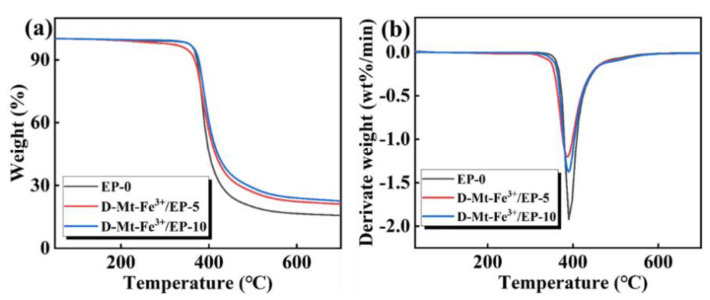
(**a**) TGA and (**b**) DTG of EP-0 and D-Mt-Fe^3+^/EP.

Epoxy resin with an excellent thermal stability is crucial for fire-retardancy application. The thermal stability and catalytic charring capability of D-Mt-Fe^3+^/EP composites were investigated by thermogravimetric analysis (TGA). As shown in Figure 3a,b, there is just one peak for all samples, indicating a single decomposition process with decomposition temperatures ranging from 300 °C to 450 °C. However, the char residue of D-Mt-Fe^3+^/EP-10 increased from 16% of EP-0 to 24% at 600 °C, indicating that D-Mt-Fe^3+^ significantly increases the char residue of EP during the process (detailed char residue of D-Mt-Fe^3+^/EP are shown in Table 1). This is inseparable from the catalytic charring role played by Fe^3+^ in this process [43]. It can be clearly seen from the DTG curves that the maximum weight-loss rate of D-Mt-Fe^3+^/EP-10 decreased from −1.9%/min of DPB/EP-0 to −1.2%/min, which was due to the carbon layer formed by the catalytic carbonization ability of D-Mt-Fe^3+^ to protect the matrix from destruction.

### 2.3. Flame Retardancy

**Figure 4:**
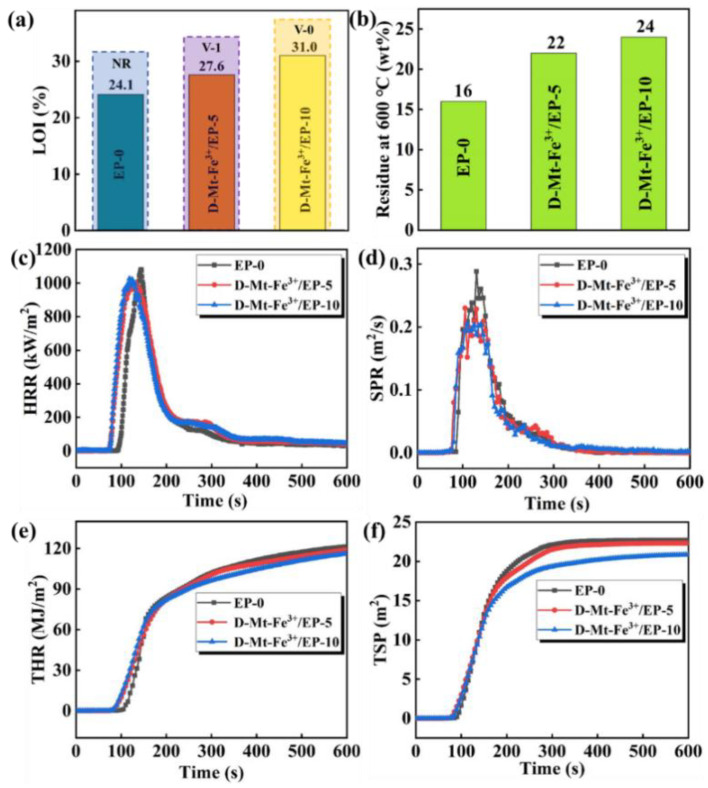
(**a**) LOI and UL-94, (**b**) residue at 600 °C, (**c**) HRR, (**d**) SPR, (**e**) THR, and (**f**) TSP of D-Mt-Fe^3+^/EP.

The vertical burning test (UL-94) and limiting oxygen index (LOI) values are the main methods used to study the flame retardancy of polymer materials. The flame retardancy of EP composites with the introduction of D-Mt-Fe^3+^ was systematically investigated using these methods. As shown in Figure 4a, the LOI values increased from 24.1% to 27.6% and 31.0%, respectively, which suggests that the LOI can be effectively improved by the addition of D-Mt-Fe^3+^. In addition, D-Mt-Fe^3+^/EP showed a significantly improved self-extinguishing ability with the rating of UL-94 testing increasing from V-1 of D-Mt-Fe^3+^/EP-5 to V-0 of D-Mt-Fe^3+^/EP-10. D-Mt-Fe^3+^/EP shows low flammability in LOI and UL-94, which is primarily due to the radical scavenging and catalytic carbonization of D-Mt-Fe^3+^.

Considering that LOI and UL-94 are small-scale laboratory simulations, there are still significant differences for the actual fire scene. The cone calorimetry test has been introduced as an improved method to evaluate the combustion behavior of D-Mt-Fe^3+^/EP. In order to investigate the flame-retardant performance of D-Mt-Fe^3+^/EP in depth, cone calorimetric tests were carried out. The heat release rate (HRR), total heat release (THR), total smoke production (TSP) and smoke production rate (SPR) are shown in Figure 4c–f. The reduced HRR of both D-Mt-Fe^3+^/EP-5 and D-Mt-Fe^3+^/EP-10 are due to the accumulation of a carbon layer during combustion. The carbon layer acts as a physical barrier, slowing down the transport of oxygen and volatiles, thereby effectually reducing the heat-release rate during the combustion process. Figure 4d shows the smoke production rate of D-Mt-Fe^3+^/EP; it can be observed that the smoke production rate decreased by 31.0% after the addition of D-Mt-Fe^3+^, indicating that D-Mt-Fe^3+^ has a significant effect on SPR reduction. Figure 4e is the THR of D-Mt-Fe^3+^/EP, which shows a trend in line with the HRR. The average effective heat of combustion (Av-EHC) is an indicator of flame suppression in the vapor phase [44]. As shown in Table 2, D-Mt-Fe^3+^/EP-5 and D-Mt-Fe^3+^/EP-10 exhibit a lower Av-EHC value than EP, indicating that D-Mt-Fe^3+^ acts as a flame suppressor in the vapor phase. That is because D-Mt-Fe^3+^ acts in the gas phase by eliminating adjacent H· and OH· radicals [45,46], thus promoting the quench of the flame. Based on the analysis above, D-Mt-Fe^3+^ plays a primary flame-retardant in the vapor phase. The mass loss rate (MLR) reflects the degree of material combustion. The MLR values of D-Mt-Fe^3+^/EP also decreases with the increase in D-Mt-Fe^3+^, indicating that the addition of D-Mt-Fe^3+^ could improve the incomplete combustion of EP. Figure 4f shows the total smoke production for D-Mt-Fe^3+^/EP and it can be observed that the total smoke production of D-Mt-Fe^3+^/EP gradually decreases following the addition of D-Mt-Fe^3+^. Meanwhile, the changes in the average carbon monoxide yield (Av-COY) and average carbon dioxide yield (Av-CO_2_Y) values further confirmed the gas-phase effect of D-Mt-Fe^3+^. The combustion chain reaction is inhibited by the trapping radicals in the gas phase by D-Mt-Fe^3+^. Therefore, less complete combustion products (CO_2_) and more incomplete combustion products (CO) are generated during the combustion process.

**Figure 5:**
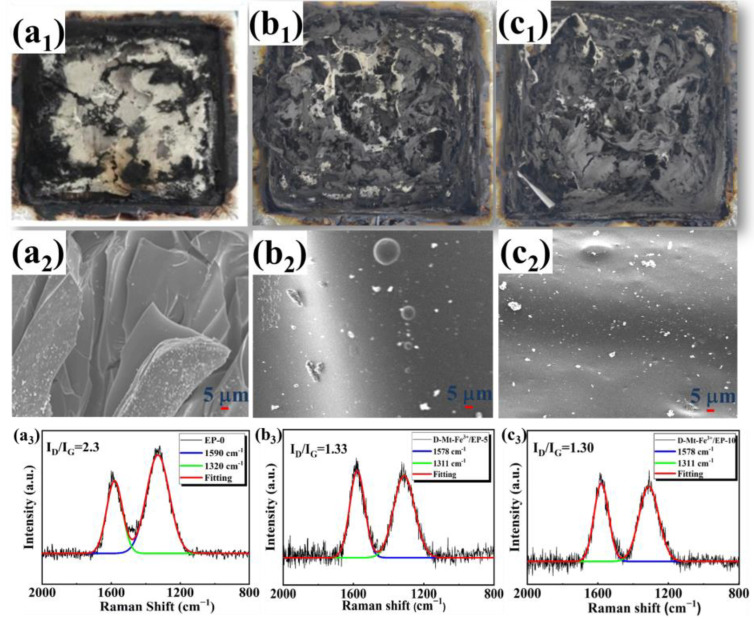
Digital photographs, SEM images and Raman spectra of residues for (**a_1_**–**a_3_**) EP-0, (**b_1_**–**b_3_**) D-Mt-Fe^3+^/EP-5 and (**c_1_**–**c_3_**) D-Mt-Fe^3+^/EP-10.

The carbon layer produced during combustion acts as a barrier to heat and oxygen, thus protecting the underlying material from damage. Therefore, the analysis of the carbon layer is crucial. In order to better investigate the char layer, the digital images and SEM of D-Mt-Fe^3+^/EP after cone calorimetry test were analyzed. As shown in Figure 5a_1_, it can be obviously found that EP-0 has almost no char remains after the cone test. From the char residues of D-Mt-Fe^3+^/EP-5, it can be found that the incorporation of D-Mt-Fe^3+^ can effectively promote the formation of a steady char structure, which is related to the catalytic carbon formation capacity of Fe^3+^ [47]. Moreover, when the loading of D-Mt-Fe^3+^ increases to 10 phr, the char layers are more complete, which can protect the underlying materials from further degradation efficiently. The SEM images of the char of D-Mt-Fe^3+^/EP-10 show a denser morphology which hinders flammable gas and oxygen transfer, thereby effectively interrupting the combustion cycle.

Raman spectra are an effective means to characterize residual carbon. As illustrated in Raman spectra (Figure 5a_3_–c_3_), there are two obvious overlapping peaks at 1311 cm^−1^ (D band) and 1578 cm^−1^ (G band), which are attributed to amorphous and graphitized carbons, respectively. The intensity ratio (I_D_/I_G_) between the D and G peaks is used to analyze the degree of graphitization of the carbon residues [48]. The smaller the D to G peak integral strength ratio (I_D_/I_G_), the higher the degree of graphitization of the carbon layer, and the stronger the corrosion resistance and heat resistance [49,50]. The value of I_D_/I_G_ for EP-0 was 2.30, while the I_D_/I_G_ for D-Mt-Fe^3+^/EP-5 and D-Mt-Fe^3+^/EP-10 were 1.33 and 1.30, respectively. It is noteworthy that the I_D_/I_G_ values for D-Mt-Fe^3+^/EP were smaller than that of EP-0, which indicates the higher degree of graphitization of D-Mt-Fe^3+^/EP. The result suggests that the D-Mt-Fe^3+^ increases the graphitic phase in char residues, which provides more effective barriers against mass and heat diffusion.

**Figure 6:**
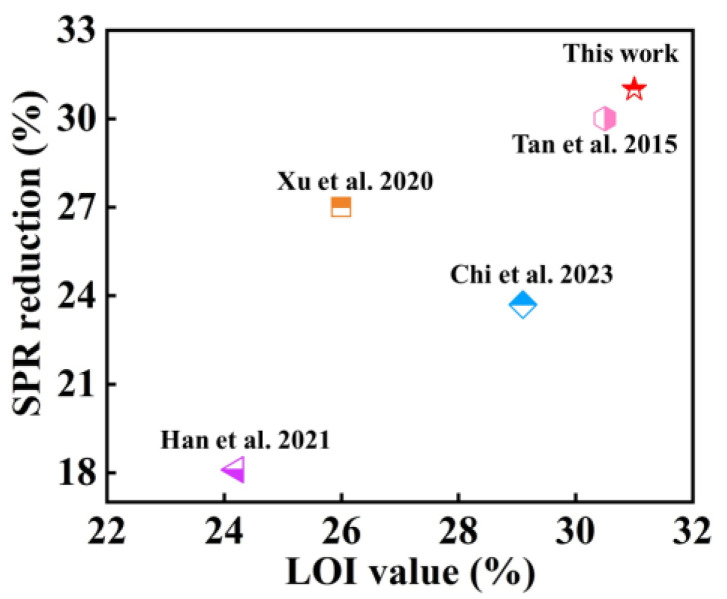
Comparison of the SPR reduction value of D-Mt-Fe^3+^/EP-10 and its corresponding LOI value with previously reported flame-retardant epoxy resins [51,52,53,54].

As shown in Figure 6, when comparing the SPR reduction value of D-Mt-Fe^3+^/EP-10 and its corresponding LOI value with the other literature on flame-retardant epoxy resins [51,52,53,54], it was found that the D-Mt-Fe^3+^ prepared in this paper showed a more outstanding comprehensive performance related to improved LOI values and the reduction in SPR. Based on the results and analysis of the above tests, the flame-retardant mechanism of D-Mt-Fe^3+^ in EP composites is as follows. In the gas phase, D-Mt-Fe^3+^ is able to prevent the combustion chain reaction by trapping H• and OH• radicals. This in turns leads to a significant reduction in the yield of CO_2_ and a reduction in TSP, which facilitates flame extinction. In the condensed phase, Fe^3+^ in D-Mt-Fe^3+^ plays a role in catalytic charring during the combustion process, forming a dense and stable carbon layer structure. On the one hand, it can hinder the release of combustible gases during the decomposition process. On the other hand, it can also block the transmission of oxygen and heat, so as to protect the substrate from being destroyed.

### 2.4. Mechanical Properties of D-Mt-Fe^3+^/EP

**Figure 7:**
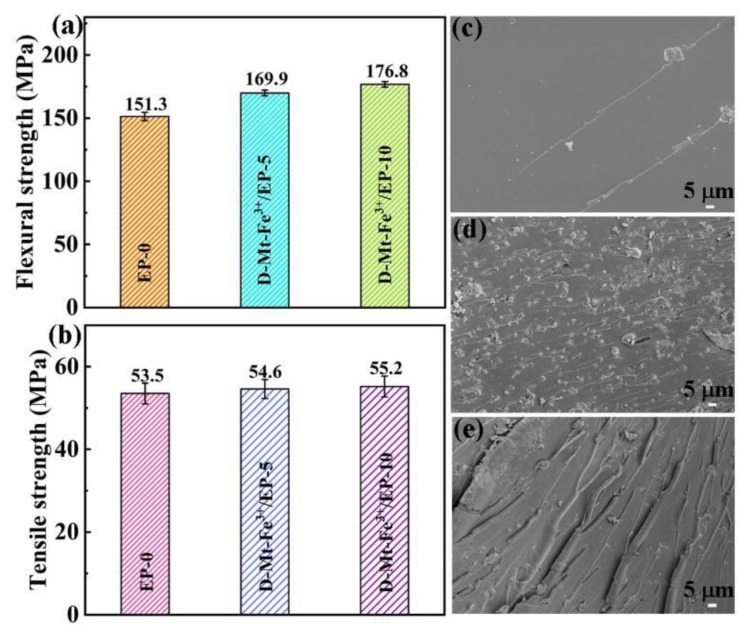
(**a**) Flexural strength, (**b**) tensile strength and SEM images of fracture surface (EP-0 (**c**), D-Mt-Fe^3+^/EP-5 (**d**), D-Mt-Fe^3+^/EP-10 (**e**)) for D-Mt-Fe^3+^/EP.

In many cases, the addition of flame retardants to improve the flame retardancy of epoxy resins often reduces its mechanical properties. Therefore, its mechanical properties are an important parameter to evaluate the comprehensive properties of resin materials. Figure 7a,b show the flexural strength and tensile strength of D-Mt-Fe^3+^/EP, respectively. Compared to EP-0 (151.3 ± 3.3 MPa), the addition of D-Mt-Fe^3+^ increases the flexural strength of EP, reaching 169.9 ± 2.3 MPa for D-Mt-Fe^3+^/EP-5 and 176.8 ± 2.2 MPa for D-Mt-Fe^3+^/EP-10. The flexural strength of D-Mt-Fe^3+^/EP-10 was increased by 25.5% compared to EP-0. The tensile strength of EP-0 was 53.5 ± 2.5 MPa, while D-Mt-Fe^3+^/EP-10 shows the best tensile properties, reaching 55.2 ± 2.5 MPa. This indicates that the tensile strength of D-Mt-Fe^3+^/EP also increases significantly with the increase in D-Mt-Fe^3+^ content.

As shown in Figure 7c, EP-0 has a smooth fracture with only a few radial streaks, indicating a typical brittle fracture due to the insufficient dispersion of stress and rapid crack extension. Unlike EP-0, D-MT-Fe^3+^/EP has distinct ductile fracture characteristics. For the representative fracture surface of D-MT-Fe^3+^/EP-10 (Figure 7e), it showed a significant increase in folds and ripples, and the direction of the ripples was perpendicular to the direction of the force, indicating that the cracks can effectively prevent the rapid fracture of the material [55].

## 3. Experimental Procedure

### 3.1. Materials

Montmorillonite (MMT) was obtained from Adamas Reagents Co., Ltd. (Shanghai, China). Iron (III) chloride hexahydrate (>99.0%, FeCl_3_·6H_2_O) was purchased from Sinopharm Chemical Reagent Co., Ltd. (Shanghai, China) Dopamine hydrochloride (>99.8%, C_8_H_12_ClNO_2_) was provided by Yitai Technology Co., Ltd. (Wuhan, China). Anhydrous ethanol (A. R., C_2_H_6_O) was supplied by Titan Technology Co., Ltd. (Shanghai, China). Tris(hydroxymethyl)aminomethane (>99.9%, C_4_H_11_NO_3_, Tris) was purchased from Macklin Biochemical Co., Ltd. (Shanghai, China). 4,4-diaminodiphenylmethane (98%, C_13_H_14_N_2_, DDM) was supplied by Sarn Chemical Technology Co., Ltd. (Shanghai, China). Bisphenol-A epoxy resin (E-51) was purchased from Fujian Yunsen Technology Co., Ltd. (Zhangzhou, China). All materials were used directly without further purification.

### 3.2. Preparation of Iron-Loaded Polydopamine Functionalized Montmorillonite (D-Mt-Fe^3+^)

Firstly, 20 g MMT was dispersed in 1 L deionized water and stirred for 12 h, then tris(hydroxymethyl)aminomethane (Tris) (2.42 g, 20 mmol) was added to adjust the pH of the solution to 8.5. A weight of 161.5 mg (0.85 mmol) dopamine hydrochloride was then added, stirred for 2 h and centrifuged at 4000 rpm for 15 min to remove the supernatant. The supernatant was removed and washed with deionized water to remove the uncoated polydopamine to obtain dopamine-modified montmorillonite (D-Mt). After that, 0.076 g (0.28 mmol) FeCl_3_·6H_2_O was added to the above mixture (the molar ratio between dopamine hydrochloride and Fe^3+^ was 3:1). The reaction mixture was continuously stirred at room temperature for 2 h. The mixture was centrifuged at 4000 rpm for 15 min and the lower layer was washed three times with deionized water to remove the uncomplexed Fe^3+^. Finally, the product was dried in an oven at 60 °C for 24 h to obtain iron-loaded polydopamine functionalized montmorillonite (D-Mt-Fe^3+^). The preparation process of D-Mt-Fe^3+^ is shown in Figure 1b.

### 3.3. Preparation of Iron-Loaded Polydopamine Functionalized Montmorillonite/Epoxy Resin Composites (D-Mt-Fe^3+^/EP)

Firstly, 5 g D-Mt-Fe^3+^ was dispersed in 30 mL anhydrous ethanol with ultrasonic oscillation for 30 min. Secondly, 100 g EP was added to this suspension with continuously stirred for 30 min to mix the EP well with D-Mt-Fe^3+^. After then, 25 g 4,4′-diamino-diphenylmethane (DDM) was added to the mixture and stirred well until the DDM was completely melted and the mixture became uniform with a stirring temperature of 100 °C. Finally, the resulting EP composite was poured into the molds and cured at 120 and 150 °C for 2 h, respectively, to obtain the cured sample. The preparation process of pure EP was similar to that of the EP composites. EP with 0, 5 and 10 phr of D-Mt-Fe^3+^ were labeled as EP-0, D-Mt-Fe^3+^/EP-5 and D-Mt-Fe^3+^/EP-10, respectively.

### 3.4. Measurements

The FTIR spectroscopy of the flakes (MMT, D-Mt and D-Mt-Fe^3+^ were mixed with KBr and pressed into flakes, respectively) was investigated using a Nicolet 5700 FTIR spectrophotometer (Nicolet, Florence, WI, USA) with 32 scans, and the wavenumber range was from 400 to 4000 cm^−1^.

Thermogravimetric analysis (TGA) was performed using an SDTA 851e thermogravimetric analyzer (Mettler Toledo, Greifensee, Switzerland). The heating rate was 10 °C/min, from 25 to 700 °C in a nitrogen atmosphere with a nitrogen flow rate of 50 mL/min.

The limiting oxygen index (LOI) was obtained using a HC-2C oxygen index instrument (Jiangning, China) in accordance with GB/T2406.2-2009 standard, and the specimen dimension was 130.0 × 6.5 × 3.0 mm^3^.

The vertical burning test (UL-94) was in accordance with the GB/T2408-2008 standard, using a CZF-2 horizontal and a vertical burning tester (Jiangning, China). The specimen dimension was 130.0 × 13.0 × 3.0 mm^3^.

The combustion test was measured using a cone calorimeter (Fire Testing Technology, East Grinstead, UK) in accordance with ISO 5660. The specimen dimension was 100.0 × 100.0 × 3.0 mm^3^ and the heat radiation value was 35 kW/m^2^.

Scanning electron microscopy (SEM) images of char residues and fracture surfaces were obtained by a JEOL-4800 Scanning Electron Microscope at an accelerating voltage of 5 kV to reveal the microscopic structure.

X-ray photoelectron spectroscopy (XPS) test was completed using an ESCALAB 250XI electron spectrometer (Thermo Fisher Scientific, San Diego, CA, USA), using Al Ka radiation (hν = 1486.6 eV) as the excitation source.

Tensile strength and flexural strength were tested using a CMT4104 universal testing machine (SANS, Rockville, MD, USA). The standard of GB/T1040.2-2006 was used for the tensile test while flexural properties were tested according to GB/T9341-2008. The load rate was 2 mm/min with five repetitions for each scale of the sample.

X-ray diffraction (XRD) patterns were scanned using a X’ Pert PXRD X-ray diffractometer (PAN alytical, Almelo, The Netherlands). The p-XRD apparatus used Cu Kα radiation (λ = 1.542 Å)-emitted X-rays which were received by a Lynx Eye detector.

## 4. Conclusions

In this study, a novel environmentally friendly flame-retardant, iron-loaded polydopamine functionalized montmorillonite was developed by a simple design. Combining the efficient ability of polydopamine to scavenge radicals with the catalytic charring effect of iron ions, the combustion risk of D-Mt-Fe^3+^/EP can be significantly reduced and its flame retardancy can be improved. Remarkably, the LOI value of D-Mt-Fe^3+^/EP-10 is 31.0% and the UL-94 test rating is V-0, which is attributed to the synergistic effect of the gas phase and the condensed phase. In the gas phase, the ability of polydopamine to trap radicals interrupts the chain reaction of combustion, resulting in a 31.0% reduction in the smoke production. In the condensed phase, the catalytic charring ability possessed by Fe^3+^ results in a denser and more intact carbon layer, which facilitates the blocking of oxygen and heat transport, thus reducing the possibility of fire risk. At the same time, the mechanical properties of D-Mt-Fe^3+^/EP are also improved with the addition of D-Mt-Fe^3+^, and the flexural strength of D-Mt-Fe^3+^/EP-10 increases by 25.5%. This work provides a convenient method for the preparation of environmentally friendly, sustainable bio-based flame retardants for epoxy resins, which offers great potential for popular advanced engineering applications such as construction materials, electrical, electronics and transportation, etc.

## Figures and Tables

**Table 1:** Relevant data from TGA and DTG tests.

Sample	T5% (°C)	Tmax (°C)	RC600 (wt%)
EP-0	370	390	16
D-Mt-Fe^3+^/EP-5	350	386	22
D-Mt-Fe^3+^/EP-10	367	389	24

**Table 2:** Cone data of D-Mt-Fe^3+^/EP.

Sample	TTI (s)	Av-EHC (MJ/kg)	MLR (g/s)	TSP (m^2^)	Av-COY (kg/kg)	Av-CO_2_Y (kg/kg)
EP-0	87	26	0.053	22.7	0.16	8.9
D-Mt-Fe^3+^/EP-5	74	25	0.050	22.3	0.46	4.2
D-Mt-Fe^3+^/EP-10	74	23	0.049	20.8	0.42	4.5

## Data Availability

Not applicable.
